# Combined Cognitive and Exercise Training Enhances Muscular Strength and Endurance: A Pilot Study

**DOI:** 10.3390/neurosci6030063

**Published:** 2025-07-14

**Authors:** Alexandru Rautu, Jesús Díaz-García, Christopher Ring

**Affiliations:** 1 School of Sport, Exercise & Rehabilitation Sciences, University of Birmingham, Birmingham B15 2TT, UK; 2 Department of Psychology, University G’ d Annunzio, 66013 Chieti-Pescara, Italy; 3 BIND-Behavioral Imaging and Neural Dynamics Center, University G’ d Annunzio, 66013 Chieti-Pescara, Italy

**Keywords:** combined training, endurance, resistance exercise, strength

## Abstract

Background: Combined cognitive and exercise training improves exercise endurance, including submaximal muscular endurance. Its effects on maximal muscular strength have yet to be determined. Accordingly, we tested the effects of combined training on muscular strength (one repetition maximum, 1RM) and endurance (as many repetitions as possible, AMRAP). Methods: Resistance-trained adults (five males, three females) completed ten sessions (four testing, six training) over 4 weeks. In each testing session, they were assessed for bench press 1RM before they completed AMRAP at 50% of initial 1RM. In each training session, they performed five bench press sets (five repetitions at 80% current 1RM), with each set followed by a hard 5 min cognitive task (Time-Load Dual-Back or Color Multi-Source Interference). Ratings of perceived exertion (RPE) were averaged to provide a session RPE. At the end of each session, participants completed a Psychomotor Fatigue Threshold Test and rated mental fatigue. Results: ANOVAs (four testing sessions) showed that combined training increased 1RM (*p* < 0.001; averaging 8.0 kg or 11% from sessions 1–4) and AMRAP (*p* < 0.01; 5.1 repetitions or 22%). Moreover, training increased RPE (*p* < 0.05; 0.3 or 5%) and decreased mental fatigue ratings (*p* < 0.001; −1.2 or −49%) but did not affect Psychomotor Fatigue Threshold Test reaction times (*p* > 0.05; 2 ms or 0%). Conclusions: A 4-week training program that combined high-intensity cognitive and resistance exercise tasks improved maximal and submaximal resistance exercise performance. This pilot study provides preliminary evidence that high-intensity combined training can enhance muscular strength and endurance.

## 1. Introduction

Grounded in motivational intensity theory [1], the psychobiological model of endurance exercise [2,3] identifies ongoing perceived effort (motivational intensity) and maximum tolerable effort (potential motivation) as determinants of endurance performance. Mental fatigue, a psychobiological state induced by long and/or hard cognitive demands, can increase perceived effort and consequently, by altering pacing, reduce endurance exercise performance [4,5].

The psychobiological mechanisms underlying resistance exercise, especially for maximal strength tasks, remain poorly understood. The limited research evidence shows that submaximal performance is vulnerable to psychobiological states, with the extent of this vulnerability varying with exercise intensity [4]. For example, mental fatigue reduced muscular endurance, measured by repetitions to failure, when participants repeatedly lifted weights corresponding to low (50% 1RM; 10.8% reduction) and moderate (70% 1RM; 13.0% reduction) but not high (90% 1RM; 6.6% reduction) intensities [6]. The most likely explanation is that low-to-moderate intensity exercise depends more on pacing than high-intensity exercise [7]. However, because the number of repetitions that can be completed at 90% 1RM is very low (c. 3–5), there is limited opportunity for psychobiological factors, such as mental fatigue, to influence pacing (cf. [8,9]). Thus, because pacing is not present in high-intensity resistance exercise, to avoid any floor effect on repetitions, another form of assessment (e.g., absolute weight lifted) is required to determine the effects of psychobiological factors on strength performance. Such assessment should reflect the neuromuscular output-reliant nature of high-intensity exercise [10].

Brain Endurance Training (BET), a form of combined cognitive and exercise training, was developed to enhance endurance exercise by repeatedly exposing individuals to concurrent low-to-moderate intensity cognitive and exercise tasks [11]. Training studies show that BET enhances subsequent aerobic endurance [12,13,14] and muscular endurance [15,16,17,18]. These studies provide evidence that BET improves endurance by decreasing perceived effort and increasing mental fatigue resilience. To date, no study has examined the effect of combined training on muscular strength.

Addressing this methodological gap requires a new and pragmatic training protocol. Thus, both the physical and cognitive training doses were increased to produce a substantial neuroplastic stimulus for developing muscular strength. The protocol considered the complexity, intensity, and duration of both physical and cognitive tasks. We reasoned that a sufficient physical dose required a neuromechanically complex and demanding exercise (bench press) being performed several times (five sets of five repetitions separated by 5 min rest) at high intensity (80% 1RM) [19,20]. Similarly, we reasoned that a sufficient cognitive dose required a battery of hard tasks involving multiple cognitive executive operations (i.e., response inhibition, memory updating, task switching, sustained attention) [21,22,23] being performed during the 5 min inter-set intervals. Thus, by implementing this repeated, intermixed exposure to high-intensity exercise and cognitive tasks, the present study was designed to evaluate the effects of combined training on muscular strength.

Evidence shows that resistance training can elicit a flow state [24,25] and that combined cognitive and physical training induces transient hypo-frontality, a temporary suppression of prefrontal cortex activity that facilitates flow [26,27]. Therefore, to understand our novel training protocol, we measured core flow features [28], namely, action-awareness merging (i.e., automaticity), concentration on task (i.e., attention), transformation of time (i.e., temporal distortion), and enjoyment [27,29]. Finally, to characterize the cognitive state induced by training, we measured core psychobiological model factors, namely, effort (i.e., RPE and repetitions in reserve, RIR) and mental fatigue (i.e., subjective ratings and reaction time).

Our study purposes were twofold. The first study purpose was to determine the effects of high intensity combined cognitive and exercise training on muscular strength and endurance. It was hypothesized that high intensity combined training would improve bench press 1RM and AMRAP at 50% 1RM. The second study purpose was to explore the acute effects of combined training on psychobiological processes. It was hypothesized that combined training would create a state of cognitive overload (increased perceived effort and mental fatigue) and increased flow.

## 2. Methods

### 2.1. Participants

Eight (five male, three female) athletes were recruited from sport and exercise science degree programs at our university. They participated in a variety of individual (e.g., martial arts, trampolining) and team (e.g., rowing, rugby) sports. Their mean (*SD*) age was 19.88 (1.96) years, mass was 70.85 (10.89) kg, and predicted 1RM was 77.63 (35.66) kg. They had at least one year (*M* = 3.38, *SD* = 1.27 years) of organized resistance training experience and were currently resistance training. They were instructed to avoid upper body resistance exercise during the study but told they could perform other forms of training if they wished. Compliance was checked during the study. They were asked to refrain from consuming caffeine and alcohol for at least 12 h before each session and to sleep at least 7 h the night before. The study protocol (ERN1867) was approved by the University of Birmingham ethics committee. Participants gave written informed consent in accordance with the Declaration of Helsinki. They were naïve to our study aims and hypotheses and were not told the weights they lifted.

### 2.2. Measures

Mental Fatigue. Subjective and behavioral measures of mental fatigue were recorded. Participants rated mental fatigue using a Borg CR10 scale, with anchors of 0 (no mental fatigue at all), 5 (substantial mental fatigue), and 10 (maximal mental fatigue). Participants completed a Psychomotor Fatigue Threshold Test (PFTT) [30] using the SOMA-NPT app (Soma Technologies). In this 90 s choice reaction time task, they were presented with 25 visual stimuli (10 green circles, 10 red circles, 5 yellow circles) on a screen and responded as quickly and accurately as possible by tapping the left/right button when they saw a green/red circle but ignored a yellow circle. Mean reaction time was computed as a behavioral measure of mental fatigue.

Rating of Perceived Exertion (RPE). Participants rated perceived exertion using a Borg CR10 scale [31] with anchors of 0 (no physical exertion at all), 5 (hard), and 10 (maximal physical exertion). The ratings in each testing session were averaged to create a session RPE.

Flow State. Participants rated items from the Flow State Scale-2 [32,33,34] on a 7-point scale with anchors of 1 (not true at all), 4 (somewhat true), and 7 (very true) to measure three dimensions of flow. Two items from each subscale assessed each dimension: action-awareness merging (“I made the correct movements without thinking about trying to do so”, “I did things spontaneously and automatically without having to think”), concentration on task (“It was no effort to keep my mind on what was happening”, “I was completely focused on the task at hand”), and transformation of time (“I felt like time went by quickly”, “I lost my normal awareness of time”). The two ratings for each subscale were averaged to yield measures of action-awareness merging, concentration on task, and transformation of time. Participants also rated two items (“I found the task enjoyable”, “It was important for me to do well on this task”) from the Intrinsic Motivation Inventory [35] on a 7-point scale with anchors of 1 (not true at all), 4 (somewhat true), and 7 (very true). The two ratings were averaged to yield a measure of enjoyment, another feature of the flow state.

Barbell movement kinematics. A draw wire displacement encoder (Model 8, East + Full) was mounted on a metal block and positioned on the floor below the bar. The encoder’s spring-loaded wire was extended and secured to the side of the barbell using a metal clamp. The encoder’s signal was recorded using a Power1401 (CED) and computer running Spike2 (CED) and digitized at 2500 Hz with 16-bit resolution. A bespoke program calculated the kinematics of each repetition. The signal for the upwards movement of each repetition (i.e., from chest to arms extended) was scored for velocity (mean velocity, m/s; maximum velocity, m/s) and the time taken to complete each upward movement (press duration, ms). In addition, the time from the end of one repetition (i.e., arms extended) until the start of the next repetition (i.e., barbell on chest) was computed (inter-repetition delay, ms).

### 2.3. Resistance Exercise Tests

One Repetition Maximum (1RM). A standard 1RM flat bench press test assessed maximal strength. In the familiarization session, the test began with a weight corresponding to 80% of predicted 1RM, and the weight was increased by 5% of predicted 1RM until the participant reached their predicted 1RM, at which point the weight was increased by 2.5% of predicted 1RM. If a lift attempt failed above 100% of predicted 1RM, the weight was decreased by 1.25% of predicted 1RM. Participants completed up to eight attempts to lift a weight. After each attempt, they provided an RPE and rested for 2 min. In subsequent testing sessions, the test began with a weight corresponding to 85% 1RM determined in the previous test and the percentage increments were based on that 1RM. The 1RM (kg) was recorded for each test. Relative 1RM was computed by dividing the 1RM by body mass.

As Many Repetitions As Possible (AMRAP). A standard AMRAP flat bench press test assessed muscular endurance [36]. Participants performed as many repetitions as possible with a weight corresponding to 50% of 1RM determined in the familiarization session. Participants were told that they could not rest between repetitions; the set was terminated if they rested. The number (*n*) of AMRAP repetitions was recorded.

### 2.4. Cognitive Training Tasks

Time-Load Dual-Back (TLDB) task. Participants completed a 5 min TLDB [17] using the SOMA-NPT app (Soma Technologies, Bloomfield, CT, USA). This very difficult executive function (i.e., memory updating, task switching, sustained attention) task switched between a primary memory updating task and a secondary decision-making task. In the 1-back task, participants were presented with a letter on screen and responded as quickly and accurately as possible by tapping the left/right arrow buttons depending on whether the letter was the same or was not the same as the previous letter. In the decision-making task, they were presented with odd/even numbers on screen and responded as quickly and accurately as possible by tapping the “1” or “2” button, respectively. The task operated in adaptive mode, with the inter-stimulus interval shortened or lengthened in the next 10-trial block if the percentage of correct responses in the current 10-trial block was 90–100 or 0–80, respectively. The starting difficulty of the task progressively increased over training sessions: 70% in sessions one and two, 80% in sessions three and four, and 90% in sessions five and six. The average post-task rating of mental fatigue was approximately 3.5 on the CR10 scale.

Color Multi-Source Interference Task (CMSIT). Participants completed a 5 min CMSIT [36] using the SOMA-NPT app (Soma Technologies). This difficult executive function (i.e., response inhibition, sustained attention) task required the identification of the correct response despite distracting and irrelevant information. Participants were presented with three numbers (1, 2, 3) in the middle of a screen in one or two sizes (small and/or large) and one or two colors (green and/or red). One of the numbers was presented twice and one of the numbers was presented once (the target). They responded by pressing as fast and as accurately as possible one of three buttons (1, 2, 3) at the bottom of the screen corresponding to the target number. They were told to ignore the colors of the numbers. The task operated in adaptive mode. The average post-task rating of mental fatigue was approximately 2.5 on the CR10 scale.

### 2.5. Procedure

Familiarization. Participants attended a familiarization session where they completed easy 1 min versions of each cognitive task, practiced using the rating scales, and received standardized instructions. For the bench press exercise, participants laid on their back on a weight bench. A strength and conditioning coach with two years of coaching experience helped participants to unrack and move the barbell to the starting position (i.e., arms fully extended). The coach supervised all testing and training sessions (see below). Each attempt (repetition) involved lowering the barbell to their lower chest (xiphisternal joint) and then pressing the barbell back to the starting position. They watched instructional videos for the warmup and were given guidance about lifting technique (“push the barbell up as fast as you can”, “keep your whole body tight”, and “squeeze your shoulder blades together”). Their body mass was recorded, they warmed up (5 min low intensity whole body cardiovascular exercise and 5 min bench press comprising 12 repetitions at 20% 1RM, 5 repetitions at 50% 1RM, and 2 repetitions at 70% 1RM) (cf. [36]), and their 1RM was determined (the starting weight was based on their predicted 1RM).

Testing. Participants completed four testing sessions on days 1 (pre-test), 11 (early-test), 21 (late-test), and 31 (post-test). Testing was scheduled for the same time of day. In each session, their body mass was recorded, they warmed up, performed 1RM, and performed AMRAP. At the end of each session, they completed a PFTT task and gave a mental fatigue rating (see Measures section). Participants rested 72 h after each testing session.

Training. Participants completed six training sessions on days 5, 8, 15, 18, 25, and 28. Testing was scheduled at the same time of day. Participants rested for 48 h after each testing session. In each session, they warmed up and performed five sets of five repetitions of the bench press at 80% 1RM (based on the previous testing session). After each set, they rated their perceived exertion and estimated their repetitions in reserve (RIR; the number of additional repetitions they believed they could have performed). Next, they performed a 5 min cognitive task, with the two tasks alternating across the five sets for 25 min of cognitive tasks per session. Two task orders (TLDB-CMSIT-TLDB-CMSIT-TLDB and CMSIT-TLDB-CMSIT-TLDB-CMSIT) were each used on three training sessions, with the same order on odd or even sessions. There was a minimal delay (<5 s) between the end of a set and the start of a cognitive task as well as between the end of a cognitive task and the start of a set. At the end of each session, they completed a PFTT task and provided ratings of mental fatigue and flow. The testing and training schedule allowed participants to rest and recover for 2–3 days between sessions.

### 2.6. Statistical Analyses

Statistical analyses were conducted using SPSS software (version 29). Significance level was set at *p* = 0.05. A 4 testing session (pre, early, late, post) ANOVA was conducted on 1RM, relative 1RM, AMRAP, PFTT reaction times, mental fatigue ratings, movement kinematics. A 3 training session block (1–2, 3–4, 5–6) with a 5 set (1, 2, 3, 4, 5) ANOVA was conducted on sessional RPE and RIR. A 3 training sessions block (1–2, 3–4, 5–6) ANOVA was conducted for measures of mental fatigue (PFTT reaction times, ratings) and flow (action-awareness merging, concentration on task, transformation of time, enjoyment). Main effects were accompanied by *t*-tests. Polynomial contrasts were used to characterize changes across the testing and training sessions. Partial eta-squared (n^2^_p_) was reported as a measure of effect size, with 0.02, 0.13, and 0.26 indicating small, medium, and large effect sizes, respectively [37].

## 3. Results

### 3.1. Tests of Muscular Strength and Endurance

**Figure 1:**
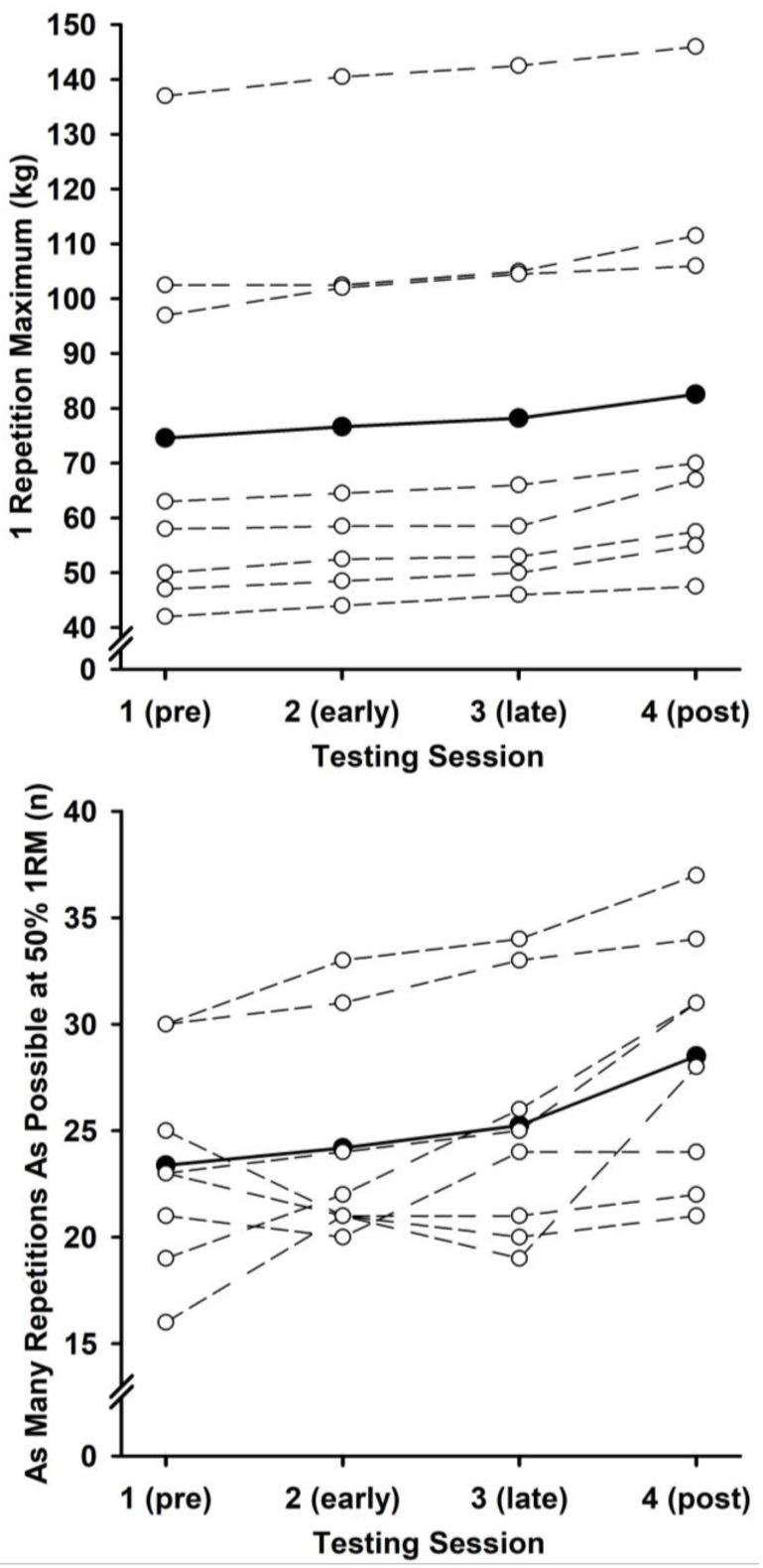
Mean (filled circles) and individual (unfilled circles) measures of muscular strength (1RM) and endurance (AMRAP) during each testing session.

A series of ANOVAs (four testing sessions) yielded large main effects for 1RM and AMRAP (Table 1). Follow-up contrast analyses confirmed strong linear trends for 1RM, *F*(1,7) = 330.50, *p* < 0.001, η_p_^2^ = 0.98, relative 1RM, *F*(1,7) = 109.27, *p* < 0.001, η_p_^2^ = 0.94, and AMRAP, *F*(1,7) = 13.43, *p* = 0.008, η_p_^2^ = 0.66. Muscular strength and endurance improved linearly across testing sessions. The group and individual 1RM and AMRAP data are shown in Figure 1.

ANOVAs found large main effects of testing session for RPE and mental fatigue rating (Table 1). RPE exhibited a strong quadratic trend, *F*(1,7) = 7.34, *p* = 0.03, η_p_^2^ = 0.51, increasing early and late testing before decreasing post testing. Mental fatigue ratings showed a strong linear trend, *F*(1,7) = 22.40, *p* = 0.002, η_p_^2^ = 0.76, decreasing linearly across the testing sessions. ANOVA detected no effect of testing session on PFTT reaction times (Table 1).

The barbell kinematics of the AMRAP repetitions are summarized in Table 1. ANOVAs revealed large main effects of testing session on the velocity and timing variables. Strong linear trends characterized the kinematics across testing sessions, with mean velocity increasing, *F*(1,7) = 7.06, *p* = 0.03, η_p_^2^ = 0.51, maximum velocity increasing, *F*(1,7) = 6.78, *p* = 0.04, η_p_^2^ = 0.49, press duration decreasing, *F*(1,7) = 14.57, *p* = 0.007, η_p_^2^ = 0.68, and inter-repetition delay decreasing, *F*(1,7) = 5.79, *p* = 0.05, η_p_^2^ = 0.45.

### 3.2. Training

**Figure 2:**
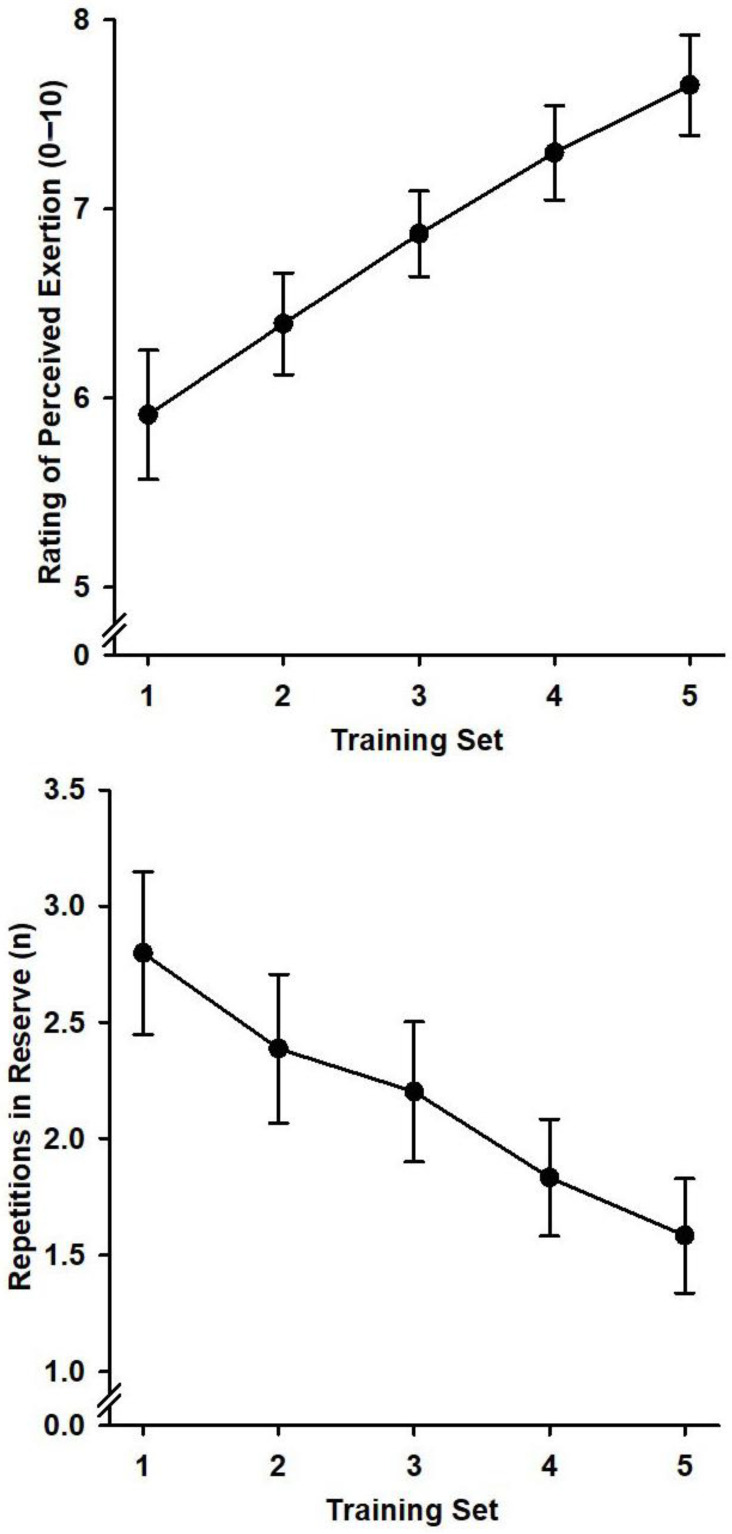
Mean (*SE*) ratings of perceived exertion (RPE) and repetitions in reserve (RIR) as a function of training set.

ANOVAs (3 training blocks by 5 sets) yielded large main set effects for RPE, *F*(4,24) = 28.33, *p* < 0.001, η_p_^2^ = 0.83, and RIR, *F*(4,24) = 19.94, *p* = 0.001, η_p_^2^ = 0.77. The group mean RPE and RIR as a function of set are shown in Figure 2. During training, RPE increased linearly, *F*(1,6) = 37.10, *p* = 0.001, η_p_^2^ = 0.86, while RIR decreased linearly, *F*(1,6) = 24.65, *p* = 0.003, η_p_^2^ = 0.80. Post hoc comparisons confirmed that RPE did not differ between set 4 and set 5, whereas RIR did not differ between sets 2 and 3. ANOVAs on barbell kinematics yielded large set effects for mean velocity, *F*(4,28) = 6.09, *p* = 0.04, η_p_^2^ = 0.47, press duration, *F*(4,28) = 4.75, *p* = 0.04, η_p_^2^ = 0.40, and inter-repetition delay, *F*(4,28) = 3.00, *p* = 0.04, η_p_^2^ = 0.30. During training, mean velocity (*M*_1…5_ = 0.28, 0.27, 0.27, 0.25, 0.25 m/s) decreased linearly, *F*(1,7) = 12.91, *p* = 0.009, η_p_^2^ = 0.65, press duration (*M*_1…5_ = 1174, 1191, 1229, 1280, 1308 ms) increased linearly, *F*(1,7) = 8.18, *p* = 0.02, η_p_^2^ = 0.54, and inter-repetition delay (*M*_1…5_ = 1058, 980, 1022, 1066, 1115 ms) decreased then increased in a quadratic pattern, *F*(1,7) = 9.89, *p* = 0.02, η_p_^2^ = 0.59. Post hoc comparisons confirmed that the mean velocities were higher and the press durations were shorter during sets 1, 2, and 3 than during sets 4 and 5. The inter-repetition delays were shorter during set 2 than sets 1 and 5 and shorter during set 3 than set 5. No effects were found for block or block by set.

The ratings, RIR, and reaction times are reported in Table 2. ANOVA (3 training session blocks) found large session main effects for PFTT reaction times, which increased linearly across training, *F*(1,7) = 5.51, *p* = 0.05, η_p_^2^ = 0.44, and enjoyment, which increased linearly across training, *F*(1,7) = 8.27, *p* = 0.02, η_p_^2^ = 0.54. The other measures did not differ across training blocks. Specifically, the data showed that training was consistently hard (high RPE, few RIR), training was not mentally fatiguing (fast PFTT reaction times, low mental fatigue ratings), and training elicited a high flow state (high action–awareness merging, high task concentration, moderate time transformation, and high enjoyment).

## 4. Discussion

This study determined the effects of four weeks of intermixed high intensity cognitive and exercise training on muscular strength and endurance. Training increased bench press 1RM by 11% and AMRAP at 50% 1RM by 22%. Mental fatigue ratings fell by −49%. Moreover, AMRAP bench press kinematics indicated that training increased mean velocity by 21%, increased maximum velocity by 18%, decreased press duration by 21%, and decreased inter-repetition delay by 23%. The training sessions induced a cognitive state characterized by low mental fatigue and high flow. Our key findings are considered below.

### 4.1. Muscular Strength and Endurance

The first study purpose was to determine the effects of combined training on resistance exercise performance. Training improved maximal muscular strength. Bench press 1RM increased by 11% from pre-test to post-test. It is hard to directly compare this finding with previous findings because the resistance training protocols (frequency, volume, intensity, exercise) vary across studies. Nonetheless, we have compared findings based on similar protocol features. First, the number of sets. Bench press 1RM increased 7% after eight weeks of training (3 sessions per week, 5 sets per session, 8–12 repetitions to failure per set, unspecified load) [36]. Second, the inter-set rest period. Bench press 1RM increased 13% for 3 min rests and 4% for 1 min rests after eight weeks of training (3 sessions per week, 3 sets per session, 8–12 repetitions to failure per set, inter-set rest 90 s, unspecified load) [38]. Third, the training load. Bench press 1RM increased 7% for 75% 1RM training loads and 2% for 40% 1RM training loads after eight weeks of training (3 sessions per week, 3 sets per session, 8–12 and 25–35 repetitions to failure per set, respectively) [39]. Thus, the current 11% improvement in 1RM compares favorably with previous study protocols.

The abovementioned comparisons support the interpretation that combined training created a strong neuroplastic stimulus and caused greater neural adaptations than expected with exercise only training. Session RPE increased 5% from pre-test to post-test whereas 1RM increased 11% from pre-test to post-test. This discrepancy between perceived effort and actual effort argues that training recalibrated the psychophysical function (i.e., how the physical stimulus was perceived) and/or increased neuromuscular efficiency (i.e., how the physical stimulus was controlled). In support of this recalibration, we noted that mental fatigue ratings decreased by 49% from pre-test to post-test. This decrease, indicative of improved fatigue resilience with training, may help account for the increase in muscular strength.

Training improved submaximal muscular endurance. AMRAP repetitions involving the same standard weight (50% initial 1RM) increased by 22% from pre-test to post-test. In this period, mean (maximum) barbell velocity increased by 21% (18%), press duration decreased by 21%, and inter-repetition delay decreased by 23%. Thus, the 22% increase in endurance (AMRAP) can be partly explained by the 11% increase in strength (1RM). The comparable increase in barbell velocity and decrease in press duration with training are indicative of increased muscular strength and efficiency and/or improved neuromuscular coordination and adaptation [40,41]. However, this explanation cannot fully account for the 22% improvement. Thus, the residual 11% of AMRAP improvement could be explained by other training factors. One factor is movement sequencing. Specifically, the reduction in inter-repetition delay reveals faster movement initiation, which is indicative of improved coordination.

Six weeks of BET improved bench press AMRAP at 40% 6RM (c. 34% 1RM) by 14% when fresh [42] and four weeks of BET improved bench press AMRAP at 80% 5RM (c. 60% 1RM) by 26% when fresh [43]. In contrast, their control groups increased AMRAP by 12% [42] and 5% [43]. These findings suggest that the greatest improvements in muscular endurance are achieved by performing hard cognitive tasks between sets. Importantly, they support the argument that the residual improvement in AMRAP observed is the current study is due to combined training.

Our findings can be compared with those from resistance training studies. Bench press AMRAP at 50% initial 1RM increased 18% after eight weeks of training (5 sets per session) [36] and bench press AMRAP at 50% 1RM decreased by 1% for 75% 1RM training loads and increased by 17% for 40% 1RM training loads after eight weeks of training [39]. Thus, we saw larger gains in muscular endurance than resistance training studies lasting twice as long. This comparison supports the argument that combined training enhances muscular endurance more than standard training. Below, we consider the training protocol to understand the performance gains.

### 4.2. Combined Training

The second study purpose was to document the acute effects of combined training on cognitive state. Participants perceived exercise to be very hard (rather than extremely hard) and exhibited very low levels of mental fatigue. They also experienced high action-awareness merging, high task concentration, high enjoyment and moderate time transformation (i.e., high flow). In sum, combined training induced a state of positive mental energy.

Perceived exertion remained relatively stable across training blocks. In each session, RPE progressively increased across sets—indicating accumulating neuromuscular fatigue—but plateaued around sets 4 and 5—contrary to typical RPE progressions [44]. A plateauing between sets 2 and 3 was observed for RIR. These non-monotonic profiles suggest a buffering effect of performing TLDB and CMSIT between sets by diverting attention and/or activating prefrontal cortical areas [7,45]. These findings suggest these tasks reduced perceived exercise intensity, recalibrating the relationship between perception of strength and actual strength. Finally, the patterning of barbell kinematics across sets suggests that the introduction of the cognitive task after the first set disrupted the normal linear development of fatigue. This disruption may help explain the changes in RPE and RIR with combined training.

Mental fatigue, indexed by ratings and reaction times, was consistently low at the end of training sessions and stable across training sessions despite 25 min of difficult cognitive tasks. It is worth noting that this was an unexpected finding that needs to be replicated and confirmed using physiological recordings. This study found that mental fatigue ratings were around 2 and PFTT reaction times were less than 600 ms. These ratings were much lower than those reported during BET [17,42]. Similarly, the PFTT reaction times were close to resting values. It is worth noting that mental fatigue was similar during training and testing (when no hard cognitive tasks were involved) and mental fatigue after training was much less than reported in previous intermixed BET studies [17] despite harder and longer cognitive tasks in the present study. This paradox may be explained by our protocol features. The first is a motivational override mechanism facilitated by task completion feedback (i.e., doing well on a hard task motivates individuals to do more) [46]. Evidence in support of this possibility comes from studies showing that exercise completion is associated with immediate reductions in self-reported mental fatigue and enhanced readiness [47]. The second (and related to the first) is a mental energy mechanism [46]. Evidence in support of this possibility comes from studies showing that exercise reduces fatigue and increases energy [48]. Mental energy has been positively linked with flow [49]. Third, a priming mechanism [50,51]. Studies show that completing a task can prime and improve the performance of a subsequent task [52].

Combined training induced a subjective high flow state. This is in line with evidence that resistance training creates a flow state [24,25]. Flow has been linked to enhanced performance. Studies show that combined demanding cognitive and exercise tasks increase perceived effort, mental fatigue and flow [26,27]. It is likely that the current combination of cognitive and physical tasks caused a transient hypofrontality that supported task performance. Replication studies using neuroimaging methods, such as near-infrared spectroscopy and electroencephalography, are needed to confirm this putative hypofrontality during high intensity combined training.

## 5. Study Limitations and Future Research Directions

This pilot study found that high-intensity combined training improved muscular strength and endurance. Any interpretations are tempered by methodological limitations. First, the study used an uncontrolled trial design. The lack of a control group makes it difficult to isolate the effects of the exercise and cognitive components of training. Without a separate physical training control group, we cannot rule out the possibility that any gains are due to physical training alone. Randomized control trials are needed to isolate the cognitive training component and identify additive or multiplicative effects. Second, the sample size was small. The low number of participants limits the generalizability and interpretability of the findings. Studies recruiting more participants are required. Third, the study incompletely described the state elicited by combined training. Studies could use subjective, behavioral, and physiological (e.g., electroencephalography, heart rate variability) measures to describe the psychobiological state. Fourth, we assessed muscular endurance using AMRAP. To explore effects of training on reserve endurance capacity, studies could ask participants to perform additional repetitions upon task failure [7]. Fifth, we tested young adult athletes with resistance training experience. The generalizability of the current findings to other age demographics and training histories needs to be explored. Finally, the study examined just one training protocol and exercise. Programmatic research can determine the optimal protocol for a range of tasks.

### Practical Applications

The results from our pilot study suggest that combined training could help optimize the performance of athletes, military personnel, and first responders. Such individuals, who frequently train and perform when fatigued and pressured, could benefit from combined training protocols (cf. [42,52]). People wishing to try out combined training should consider the complexity, intensity, and duration of the physical and cognitive tasks. We recommend repeatedly performing complex tasks at high intensities. Our protocol provides a starting point; however, the number of sets, repetitions and rest intervals as well as task intensities should be explored by athletes to identify their optimal settings.

## 6. Conclusions

High-intensity combined training—intermixing high-load resistance training and hard executive function cognitive tasks—produced substantial gains in muscular strength and endurance coupled with greater mental fatigue resilience. These gains exceeded those typically observed with resistance training. The psychobiological state elicited by combined training, namely, high flow and low mental fatigue, suggests that training created a potent stimulus for neuroplastic adaptations (e.g., improved muscular control and recalibration of effort) underlying performance. We acknowledge that the small sample size and lack of a control group are limitations of the current study. Importantly, this pilot study outlines a novel combined cognitive and physical training methodology that could be used to enhance physical performance.

## Figures and Tables

**Table 1:** Mean (95% CI) measures of muscular performance, cognitive state, and AMRAP barbell kinematics as a function of testing.

Measure		Testing Session			*F*(3,21)	*p*	η_p_^2^
	1 (Pre)	2 (Early)	3 (Late)	4 (Post)			
1RM (kg)	74.56 (46.28, 102.85)	76.63 ^a^ (47.83, 105.42)	78.19 ^a,b^ (48.97, 107.40)	82.56 ^a,b,c^ (53.55, 111.57)	58.24	***	0.89
Relative 1RM (kg/kg)	1.03 (0.75, 1.31)	1.05 ^a^ (0.77, 1.33)	1.07 ^a,b^ (0.79, 1.35)	1.14 ^a,b,c^ (0.86, 1.43)	39.32	***	0.85
AMRAP (*n*)	23.38 (19.26, 17.49)	24.19 (20.01,28.37)	25.25 (20.53, 29.97)	28.50 ^a,b,c^ (23.67, 33.33)	7.58	***	0.52
Session RPE	6.72 (6.13, 7.32)	7.26 ^a^ (6.81, 7.71)	7.24 ^a^ (6.64, 7.84)	7.06 (6.31, 7.81)	3.79	*	0.35
PFTT RT (ms)	523 (448, 598)	525 (463, 587)	535 (465, 606)	521 (443, 598)	0.06		0.01
Mental Fatigue (0–10)	2.44 (1.33, 3.54)	1.81 ^a^ (0.63, 2.99)	1.38 ^a^ (0.55, 2.20)	1.25 ^a,b^ (0.30, 2.20)	10.52	***	0.60
Mean Velocity (m/s)	0.38 (0.30, 0.45)	0.41 (0.35, 0.47)	0.43 (0.37, 0.50)	0.46 ^a^ (0.40, 0.53)	3.72	*	0.35
Maximum Velocity (m/s)	0.55 (0.47, 0.66)	0.58 (0.50, 0.66)	0.62 ^b^ (0.54, 0.70)	0.65 ^b^ (0.55, 0.75)	3.63	*	0.34
Press Duration (ms)	957 (813, 1101)	839 (768, 910)	795 ^a^ (712, 879)	755 ^a,b^ (668, 843)	6.56	**	0.48
Inter-Repetition Delay (ms)	916 (624, 1208)	879 (601, 1156)	788 (618, 957)	709 ^a^ (577, 840)	3.28	*	0.32

Note: * *p* < 0.05, ** *p* < 0.01, *** *p* < 0.001. Superscripts ^a^, ^b^, and ^c^ indicate a significant mean difference for sessions 1, 2, and 3, respectively. 1RM = one repetition maximum. AMRAP = as many repetitions as possible. RPE = rating of perceived exertion. PFTT RT = psychomotor fatigue threshold test reaction time.

**Table 2:** Mean (95% CI) measures of perceived exertion, repetitions in reserve, fatigue, barbell kinematics, and flow as a function of training.

Measure	Training Sessions (Blocks)			*F*(2,14)	*p*	η_p_^2^
	1–2	3–4	5–6			
RPE (0–10)	6.56 (5.90, 7.21)	6.88 (6.24, 7.51)	7.04 (6.31, 7.78)	2.51		0.30
RIR (*n*)	2.29 (1.50, 3.08)	2.18 (1.42, 2.94)	2.01 (1.41, 2.60)	1.88		0.24
PFTT RT (ms)	501 (439, 564)	547 ^a^ (513, 581)	567 ^a^ (500, 634)	3.73	*	0.35
Mental Fatigue (0–10)	2.19 (0.95, 3.43)	1.58 (0.54, 2.62)	1.88 (0.66, 3.09)	3.38		0.33
Mean Velocity (m/s)	0.26 (0.22, 0.30)	0.26 (0.22, 0.31)	0.26 (0.22, 0.31)	0.02		0.00
Maximum Velocity (m/s)	0.39 (0.34, 0.44)	0.38 (0.32, 0.44)	0.38 (0.31, 0.45)	0.34		0.05
Press Duration (ms)	1264 (1113, 1416)	1240 (1112, 1368)	1205 (1333, 1276)	0.52		0.07
Inter-Repetition Delay (ms)	1147 (924, 1370)	1036 (833, 1239)	962 (840, 1084)	3.28		0.32
Action–Awareness Merging (1–7)	5.50 (4.88, 6.12)	5.91 (5.18, 6.63)	6.06 (5.41, 6.71)	1.91		0.21
Concentration on Task (1–7)	5.53 (5.09, 5.97)	5.56 (4.79, 6.33)	5.84 (4.99, 6.70)	0.86		0.11
Transformation of Time (1–7)	4.66 (3.92, 5.39)	4.91 (4.25, 5.57)	4.94 (4.10, 5.77)	0.76		0.10
Enjoyment (1–7)	5.44 (4.93, 5.94)	5.63 (5.97, 6.28)	5.84 ^a^ (5.29, 6.40)	5.02	*	0.42

Note: * *p* < 0.05. Superscript ^a^ indicates a significant mean difference from session blocks 1–2. RPE = rating of perceived exertion. RIR = repetitions in reserve. PFTT RT = psychomotor fatigue threshold test reaction time.

## Data Availability

Data are available from the corresponding author upon request or via our open research repository: University of Birmingham Institutional Research Archive eData for research datasets. http://www.ubira.bham.ac.uk/, accessed on 22 June 2025.
